# Lack of Transmission among Close Contacts of Patient with Case of Middle East Respiratory Syndrome Imported into the United States, 2014

**DOI:** 10.3201/eid2107.150054

**Published:** 2015-07

**Authors:** Lucy Breakwell, Kimberly Pringle, Nora Chea, Donna Allen, Steve Allen, Shawn Richards, Pam Pantones, Michelle Sandoval, Lixia Liu, Michael Vernon, Craig Conover, Rashmi Chugh, Alfred DeMaria, Rachel Burns, Sandra Smole, Susan I. Gerber, Nicole J Cohen, David Kuhar, Lia M. Haynes, Eileen Schneider, Alan Kumar, Minal Kapoor, Marlene Madrigal, David L. Swerdlow, Daniel R. Feikin

**Affiliations:** Centers for Disease Control and Prevention, Atlanta, Georgia, USA (L. Breakwell, K. Pringle, N. Chea, M. Sandoval, S.I. Gerber, N.J. Cohen, D. Kuhar, L.M. Haynes, E. Schneider, D.L. Swerdlow, D.R. Feikin);; Indiana State Health Department, Indianapolis, Indiana, USA (D. Allen, S. Allen, S. Richards, P. Pantones, M. Sandoval, L. Liu);; Cook County Department of Public Health, Oak Forest, Illinois, USA (M. Vernon);; Illinois Department of Public Health, Chicago, Illinois, USA (C. Conover);; DuPage County Health Department, Wheaton, Illinois, USA (R. Chugh);; Massachusetts Department of Public Health, Jamaica Plain, Massachusetts, USA (A. DeMaria, R. Burns, S. Smole);; Community Hospital, Munster, Indiana, USA (A. Kumar, M. Kapoor, M. Madrigal)

**Keywords:** Middle East respiratory syndrome, MERS-CoV, coronavirus, contact tracing, infection control, United States, viruses, transmission, imported case, global positioning system tracer tags, self-reporting, contact, exposure

## Abstract

In May 2014, a traveler from the Kingdom of Saudi Arabia was the first person identified with Middle East respiratory syndrome coronavirus (MERS-CoV) infection in the United States. To evaluate transmission risk, we determined the type, duration, and frequency of patient contact among health care personnel (HCP), household, and community contacts by using standard questionnaires and, for HCP, global positioning system (GPS) tracer tag logs. Respiratory and serum samples from all contacts were tested for MERS-CoV. Of 61 identified contacts, 56 were interviewed. HCP exposures occurred most frequently in the emergency department (69%) and among nurses (47%); some HCP had contact with respiratory secretions. Household and community contacts had brief contact (e.g., hugging). All laboratory test results were negative for MERS-CoV. This contact investigation found no secondary cases, despite case-patient contact by 61 persons, and provides useful information about MERS-CoV transmission risk. Compared with GPS tracer tag recordings, self-reported contact may not be as accurate.

## Abstract 2

Despite 61 contacts with unprotected exposure, no secondary cases occurred.

Middle East respiratory syndrome coronavirus (MERS-CoV) is a lineage C betacoronavirus that was first reported in September 2012 in a patient from the Kingdom of Saudi Arabia (*1*). By September 8, 2014, a total of 837 laboratory-confirmed cases and 292 associated deaths had been reported by the World Health Organization. All reported case-patients have resided in or had recent travel to the Arabian Peninsula and neighboring countries (*2*).

Clusters of MERS-CoV infection have occurred within extended families, households, and healthcare settings (*3*–*6*). Contact investigations around imported cases in the United Kingdom, France, and Tunisia identified cases among household and healthcare contacts, suggesting person-to-person transmission (*7*–*9*). However, these investigations found limited onward transmission: a maximum of 3 second-generation cases were found among investigations with total contacts ranging from 7–163 persons (*7*–*9*). Other contact investigations of imported cases outside of the Middle East have found no secondary transmission (*10*–*13*).

On April 29, 2014, the Indiana State Department of Health (ISDH) informed the Centers for Disease Control and Prevention (CDC) of a patient under investigation for MERS-CoV infection. A clinical specimen from the patient was confirmed positive by CDC on May 2, 2014 (*5*); this infection was identified as the first imported MERS case in the United States. The case-patient, a physician and resident of Saudi Arabia, traveled by airplane to Chicago, Illinois, USA, via London, United Kingdom, then by bus to Indiana, USA. He stayed with his family in Indiana for 4 days, during which time he twice met with a business associate in Illinois before seeking medical care at an Indiana hospital; multiple healthcare personnel (HCP) at the hospital were exposed to the patient (*14*). Given the uncertainty around how MERS-CoV is transmitted, we conducted a comprehensive contact investigation of this case to characterize exposures in household, community, and hospital settings and to quantify the risk of transmission. We also compared contact reported by HCP during standardized interviews with those in global positioning system (GPS) tracer tag recordings.

## Methods

### Ethical Review

This investigation was part of a public health response, so it was determined by CDC to be a nonresearch investigation and not subject to review by the CDC Institutional Review Board. All participants provided verbal consent before interview; parental permission and assent from from minors were obtained as appropriate.

### Definitions and Identification of Contacts

For the purpose of this investigation, we defined contacts as all persons who had potential exposure to the case-patient before airborne and contact precautions were instituted. More specifically, we defined HCP contacts as all persons who had a face-to-face (within 1 meter) interaction with the case-patient or who entered the case-patient’s room without appropriate personal protective equipment (PPE; i.e., gloves, N95 respirator, gown, and eye protection) before airborne and contact precautions were instituted. HCP contacts were identified by reviewing GPS tracer tag logs, the case-patient’s medical chart, and emergency department (ED) security video footage or through the hospital hotline, on which personnel could self-identify. GPS tracer tags were worn routinely by registered nurses (RNs) and certified nursing assistants (CNAs). The tags track the date and time that staff enter and exit a patient’s room. We reviewed hospital GPS records to determine the exposure time and number of patient visits for attending RNs and CNAs.

Hospital visitor contacts were defined as all persons who visited the case-patient at the hospital before airborne and contact isolation precautions were instituted. Household contacts were defined as all persons who stayed overnight in the same household as the case-patient between his arrival in the United States and his admission to the hospital. Community contacts were defined as all persons, other than household or HCP contacts, who had face-to-face exposure to the case-patient. Hospital visitor, household, and community contacts were identified from interviews with the case-patient, family members, and hospital staff.

### Duration of Exposure, Infection Monitoring, and Quarantine

Duration of exposure was determined by asking contacts how much time they had spent with the case-patient. Duration of exposure was also calculated from GPS records.

Following confirmation (on May 2, 2014) that the patient was infected with MERS-CoV, HCP and household contacts checked their body temperature twice daily and self-monitored for respiratory or gastrointestinal symptoms for a total of 14 days after their last exposure to the case-patient. HCP also reported to the hospital’s Employee Health Services each day. In addition, nonphysician HCP contacts were requested to self-quarantine at home or wear surgical masks in the community, and physician HCP contacts were requested to wear surgical masks at work.

### Interviews

The case-patient was asked to report his medical and exposure history, health care–seeking behaviors, job-related activities, and social activities during the 14 days before illness onset. HCP, household, and community contacts answered standard questionnaires covering basic demographic information; infection control practices when in contact with the case-patient; type, length, and frequency of contacts with the case-patient; chronic medical conditions; and symptoms since first exposure to the patient.

### Biologic Specimen Collection

Serum, nasopharyngeal swab, oropharyngeal swab, stool, and urine samples were collected from the case-patient on various dates (*15*). Two sets of nasopharyngeal and oropharyngeal swab samples and serum samples were collected from all contacts. The initial and follow-up sets of specimens were collected on postexposure days 3–8 and 12–14, respectively. An additional set of specimens was collected within 48 hours from any contacts who became symptomatic.

Nasopharyngeal and oropharyngeal swab samples were tested at the ISDH laboratory, Massachusetts Department of Public Health, Illinois Department of Public Health, or CDC within 72 hours of collection. Stool and urine samples were tested at the ISDH laboratory, and serum samples were tested at CDC.

### Laboratory Testing

Nasopharyngeal, oropharyngeal, urine, serum, and stool specimens were tested by using a MERS-CoV real-time reverse transcription PCR (rRT-PCR) developed by CDC, as previously described (*15*). Serum specimens collected on postexposure days 12–14 were screened for MERS-CoV–specific IgG, IgM, and IgA by using a recombinant nucleocapsid–based ELISA. Positive ELISA results were confirmed by MERS-CoV immunofluorescence assay (IFA) and microneutralization assay (*14*). A specimen positive by ELISA, indeterminate or negative by IFA, and negative by microneutralization was determined to be negative. A positive serologic result required a positive ELISA result and confirmation by IFA or microneutralization assay. On the basis of clinical discretion, a multiplex PCR assay virus panel (Biofire Diagnostics, Salt Lake City, UT, USA) was performed on samples from the case-patient and 3 contacts.

### Data Analyses

Basic descriptive analyses were conducted for all contacts. When available, self-reported and GPS-monitored exposure time and number of visits were compared by calculating Pearson correlation coefficients.

## Results

### Case-Patient

The case-patient worked at a Saudi Arabia hospital where patients infected with MERS-CoV had been treated in April 2014. He did not recall caring for known MERS patients or patients with respiratory symptoms, but he did perform noninvasive procedures, using appropriate PPE, on 3 or 4 intubated patients. None of his colleagues, friends, or household members had respiratory symptoms during April. Beginning on April 18 (i.e., day of illness [DOI] 1), he had low-grade fever, fatigue, and myalgias. On DOI 6, he departed for the United States; on DOI 10, a mild, nonproductive cough and shortness of breath developed. The case-patient was admitted to the hospital on DOI 11 for right lower lobe pneumonia with hypoxia. On DOI 12, he was suspected of having MERS-CoV infection, so airborne precautions (i.e. N95 respirator and patient isolation in an airborne infection isolation room) were instituted. At 11:00 AM on DOI 13, after MERS-CoV infection was confirmed, contact precautions were initiated and the case-patient was moved to another airborne infection isolation room with an anteroom. Test results for sputum, oropharyngeal swab, and plasma samples continued to be positive for MERS-CoV until DOI 16. A detailed report of the case-patient’s clinical course is published elsewhere (*14*). The case-patient was discharged from the hospital 11 days after admission (DOI 21).

### Contact Investigation

#### HCP Contacts

**Figure 1:**
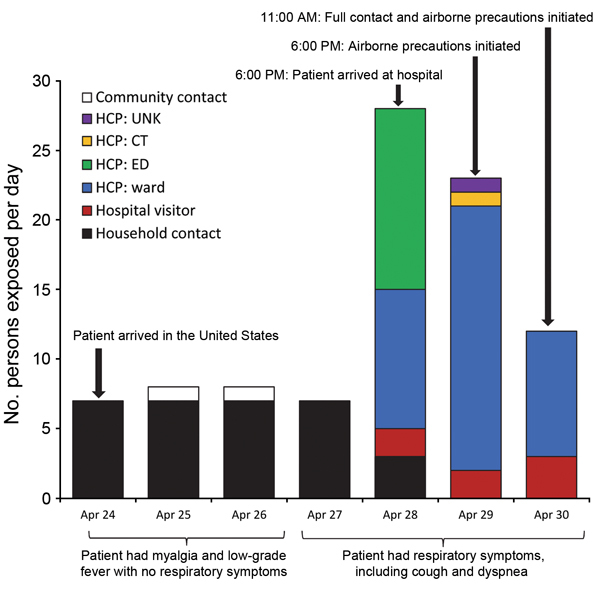
Number and type of contacts exposed to a Middle East respiratory syndrome coronavirus case-patient per day after his arrival in the United States on April 24, 2014. The same persons could be counted on multiple days of exposure. CT, computed tomography department; ED, emergency department; UNK, unknown; ward, patient’s hospital floor.

Fifty-three HCP self-identified as contacts of the case-patient or were identified as contacts from security video footage, GPS tracer tag logs, or the case-patient’s medical record. Two HCP declined to be interviewed, and 3 could not be reached. Of the 48 HCP contacts interviewed within 1 week of exposure, 3 were determined to not to be contacts and were excluded from the analyses. Of the remaining 45 HCP contacts, 23 were exposed to the patient on hospitalization day 1 (13 in the ED and 10 in the patient’s room or the computerized tomography suite), 19 were exposed on hospitalization day 2, and 9 were exposed on hospitalization day 3; several HCP were exposed on multiple days (Figure 1).

Of the 45 HCP contacts, 7 (16%) were men and 38 (84%) were women. The median age was 41.5 years (range 22.0–61.0 years). HCP in several job classifications were exposed to the case-patient, but most (47%) were RNs or CNAs. Most HCP contacts (71%) were assigned to work in the ED (n = 21 [47%]) or ward in which the case-patient was hospitalized (11 [24%]); however, 12 (27%) HCP contacts worked in multiple departments (Table).

**Table:** Demographic, employment, and exposure information for health care personnel contacts of patient with the first imported case of Middle East respiratory syndrome into the United States, 2014*

Health care personnel data	No. (%)
Sex	
M	7 (16)
F	38 (84)
Age group, y	
<30	13 (29)
30–39	9 (20)
40–65	23 (51)
Occupation	
Administration	3 (7)
Housekeeping	2 (4)
Medical doctor	3 (7)
Nurse practitioner	1 (2)
Nursing assistant	10 (22)
Phlebotomist	4 (9)
Radiology technician	4 (9)
Respiratory therapist	6 (13)
Registered nurse	11 (24)
Social personnel	1 (2)
Primary employment location in hospital	
Ward	21 (47)
Emergency department	11 (24)
Multiple locations	12 (27)
Computed tomography suite	1 (2)
Personal protective equipment worn while in contact with the patient†	
Gown	0
Goggles	2 (5)
N95 respirator	6 (14)
Surgical mask	2 (5)
Pre-existing condition‡	
Yes	4 (9)
No	40 (89)
No. self-reported times HCP visited the patient’s room between 6:00 PM April 28 and 11:00 PM April 30	
0	1 (2)
1	26 (58)
2–5	11 (24)
6–9	3 (7)
>10	4 (9)

*HCP, health care personnel; ward, patient’s hospital floor.. †Full personal protective equipment includes N95 respirator, goggles, gown and gloves. ‡Pre-existing conditions that may increase the risk of infection included current pregnancy, chronic steroid use and diabetes.

Six HCP contacts were nonclinical staff (administration, housekeeping, or social services) who had direct contact with the case-patient’s surroundings but never touched the case-patient. Thirty-three HCP contacts (physicians, RNs, CNAs, phlebotomists, and radiology technicians) touched the case-patient while performing activities such as recording vital signs, listening to his lungs, and drawing blood. RNs and CNAs had the most frequent exposures; the median number of self-reported visits for each RN and CNA were 7 and 2, respectively (Table). Six respiratory therapists touched the case-patient and administered nebulizer treatments or spirometry tests.

Because airborne precautions began ≈24 hours after admission, most HCP contacts (39 [86.7%]) did not use a respirator or surgical mask while attending to the case-patient. Four HCP contacts had underlying medical conditions (current pregnancy, diabetes, or chronic steroid use), which might increase their risk for MERS-CoV infection or disease. Most HCP contacts (26 [58%]) were exposed to the case-patient 1 time; 18 were exposed >2 times, and 4 were exposed >10 times (Table). Overall, the median total self-reported exposure time was 11 minutes 30 seconds (range 15 s to 69 min 45 s). Two HCP contacts were excluded from length and frequency of exposure analyses because they could not remember their exposure to the case-patient.

The following symptoms most commonly developed in 9 HCP contacts: rhinorrhea (33%), odynophagia (22%), or headache (22%) within postexposure day 14; more than 1 symptom developed in some contacts. Fever did not develop in any of these contacts.

#### Hospital Visitor Contacts

Three family members were identified as hospital visitor contacts: 2 were also household contacts, and the other was an out-of-town family member who had not been exposed in the household. Two of these contacts were exposed on hospitalization days 1 and 2 without wearing any PPE, and all 3 were exposed on hospital day 3 while wearing N95 masks but no other PPE (Figure 1).

#### Household Contacts

Of the 7 household contacts, 5 permanently resided in the house where the case-patient stayed in the United States, and 2 were visiting from Massachusetts. One household contact was also an HCP contact and was included in both categories. All household contacts had minimal exposure to the case-patient during DOI 7–10 because he had isolated himself during most of his stay. Three household contacts reported hugging and kissing him on the day he arrived (DOI 7) and spending a few hours in the car with him before hospital admission (DOI 7–10). Coryza, but not fever, developed in 2 household contacts; 1 of these contacts tested positive for rhinovirus.

#### Community Contact

The 1 community contact was a business associate of the case-patient. The contact shook hands with the case-patient and had 2 face-to-face meetings with him on April 25 (2.0 h in length) and April 26 (1.5 h in length). At that time, the case-patient had mild myalgias and fever without any respiratory symptoms. On May 14, the contact had a runny nose and mild cough, but fever did not develop, and the contact had test results positive for rhinovirus.

### Laboratory Results

For 60 contacts, both initial and follow-up nasopharyngeal and oropharyngeal swab samples and serum samples were negative for MERS-CoV by rRT-PCR and for MERS-CoV–specific antibodies by serologic testing. For the community contact, MERS-CoV test results for initial and follow-up nasopharyngeal and oropharyngeal swab samples and serum samples were negative by rRT-PCR, low titer–antibody positive by ELISA, indeterminate by IFA, and negative by microneutralization assay. His MERS-CoV antibody status was determined to be negative because the ELISA result could not be confirmed by either IFA or microneutralization assay. Additional nasopharyngeal, oropharyngeal, and serum samples from 8 symptomatic HCP contacts were negative for MERS-CoV by rRT-PCR and serologic testing.

### Self-Reported Versus Monitored HCP Exposure Duration and Number of Visits

**Figure 2:**
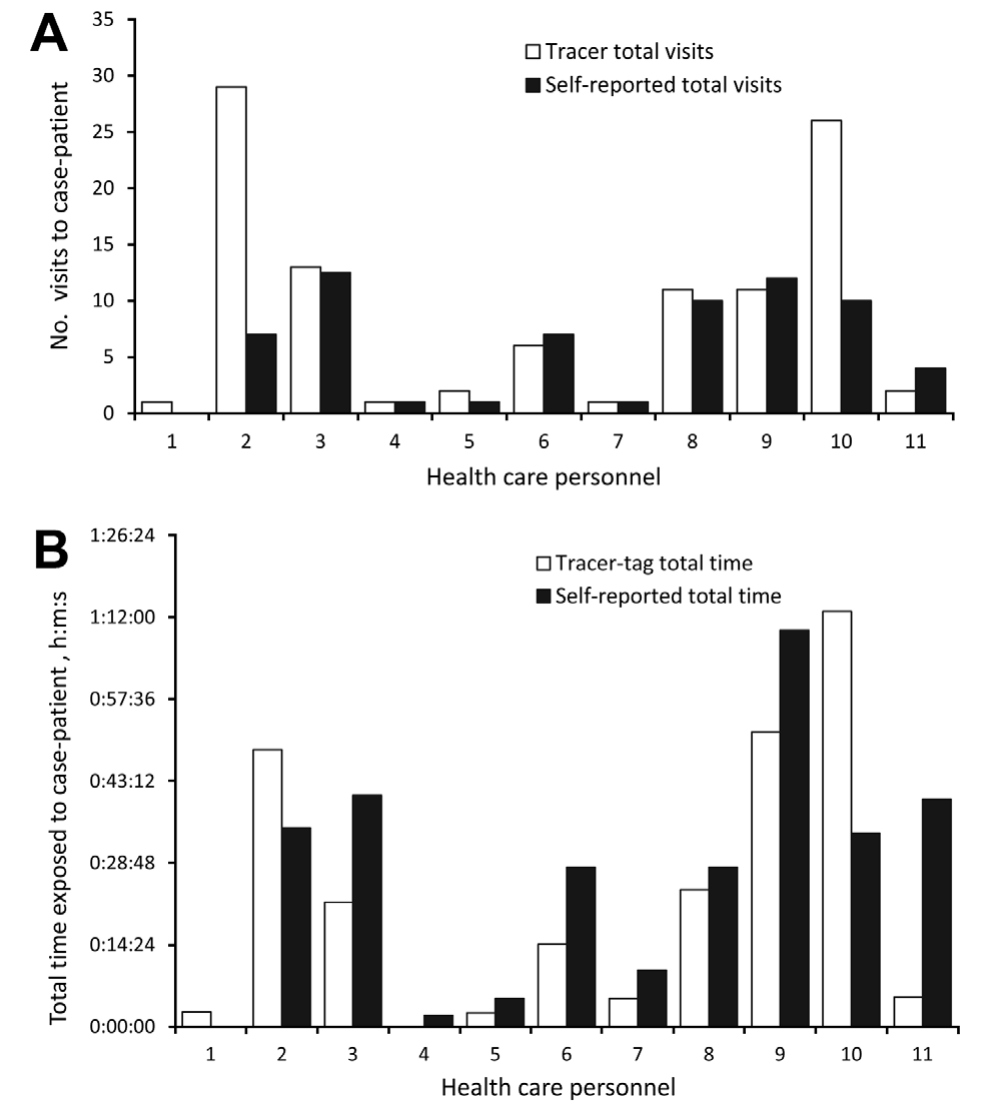
Comparison of self-reported and global positioning system (GPS) tracer tag–reported visits (A) and exposure times (B) for health care personal (HCP) who had contact with a Middle East respiratory syndrome coronavirus case-patient during his hospitalization, United States, 2014. Visits and exposures could be reported for 8 certified nursing assistants and 3 registered nurses who wore GPS tracer tags. The self-reported number of visits to the patient’s room was derived from interviews held 5–7 days after exposure to the case-patient.

Of the 45 HCP contacts, 11 (24%; 3 RNs and 8 CNAs) wore GPS tracer tags. Of those 11 contacts, 8 reported a number of visits to the patient’s room similar (±2) to that recorded by the tracer tag; 1 underestimated the number by 22 visits; 1 underestimated the number by 16 visits; and 1 did not recollect the tag-recorded visits (Figure 2, panel A). There was no consistent pattern in the way HCP reported their number of visits: some overestimated and others underestimated the number. The total exposure time was more difficult for HCP to recall. Five estimated their exposure time within 10 minutes of the tracer tag–reported time, and 4 estimated within 20 minutes (Figure 2, panel B). The maximum time difference between cumulative self-reported and tracer tag–recorded time was 39 minutes. No significant correlation was found between self-reported and GPS-measured time (R^2^ = 0.47) and number of visits (R^2^ = 0.45) with the case-patient.

## Discussion

We describe the contact investigation of the first identified MERS patient in the United States. All 61 identified contacts had negative test results for MERS-CoV even though some had face-to-face interactions with or prolonged exposure to the case-patient or administered nebulizer treatments and spirometry tests to the case-patient.

The absence of transmission to household contacts could be explained by the case-patient’s mild initial respiratory symptoms, his hospital admission <24 hours after respiratory symptom onset (DOI 11), his self-isolation at home, and his lack of need for caregiving assistance before admission, all of which served to limit household members’ exposure. Similarly, the absence of transmission to the community contact may have been due to the case-patient’s lack of respiratory symptoms during the 2 meetings. The absence of transmission to household and community contacts in this investigation is similar to that seen in contact investigations of several other patients with MERS (*11*–*13*); however, in other settings, transmission to household members who provided care to persons with MERS-CoV infection have been reported, and household clusters have been documented (*3*–*7*,*16*).

When the case-patient was admitted during the second week of illness, the virus load in his sputum was high (*14*). However, none of the HCP contacts became infected. Serologic results may become positive >10–14 days after exposure, so we minimized the possibility of missing any asymptomatic infections by combining serologic results with clinical evaluation and PCR results. The absence of transmission to the HCP contacts may have been due to the absence of high-risk procedures (e.g., intubation, respiratory suctioning, and bronchoscopy), the short duration of exposure, and the few HCP contacts with underlying medical conditions. In addition, the hospital implemented strict infection control practices soon after the case-patient was suspected of having MERS-CoV infection, limiting the number and duration of exposures. These findings are similar to those from some previously documented contact investigations (*12*). However, there have been reports of transmission to HCP contacts in hospitals with multiple MERS patients or delayed implementation of appropriate infection control practices (*5*,*7*). The different findings from reported investigations illustrate that the specific activities that lead to an increased risk for MERS-CoV transmission still need to be clearly defined.

Severe acute respiratory syndrome coronavirus (SARS-CoV) emerged in 2002 in China and spread globally in 2003. SARS-CoV shares similar characteristics with MERS-CoV, including likely zoonotic origin and transmission (*17*–*20*). Recent research on MERS-CoV has demonstrated plausibility for zoonotic transmission from dromedary camels to humans (*21*,*22*). MERS-CoV seems less able than SARS-CoV to spread from person-to-person (*23*–*26*). Reports from the SARS-CoV epidemic showed tertiary transmission to >100 people, and 20% of health care workers become infected from the index patient (*24*,*27*). Most documented clusters of MERS-CoV infection show limited spread outside certain hospital settings, and unlike transmission in the SARS-CoV epidemic, there have been no foci of sustained transmission outside of the MERS-CoV infection epicenter in and near the Arabian Peninsula (*23*). However, as with SARS-CoV, the risk for MERS-CoV transmission may vary by patient, and health care facilities must maintain a high index of suspicion and immediately institute appropriate infection control practices for suspected cases.

This investigation is unique because we had independent documentation of duration of exposure from GPS-based tracer tags for 20% of HCP contacts. Most HCP contacts accurately reported case-patient exposure. However, HCP with the most contact had poorer recall accuracy, and 20 minutes’ difference in exposure may alter the HCP contact risk, given that each visit was generally <3 minutes in duration. These findings have important implications for future contact investigations, and we recommend using objective measures of exposure, such as surveillance footage or GPS tracer tags, when available. In addition, we note that self-reported exposures are not always accurate because the accuracy of recalled time versus actual time spent with case-patients may be less reliable for HCP contacts that see a patient regularly for short periods of time. 

This investigation had some limitations. First, risk factors for transmission could not be analyzed because none of the contacts were infected. Second, the use of the GPS tracer tag system to monitor HCP interaction with the case-patient might not always have given accurate results because HCP may not have been wearing their assigned tag when entering the room or, conversely, may have stood close to but not in the room, causing the tracking system to record incorrectly that the HCP had entered the room. Use of the GPS system also does not account for changes in risk to HCP contacts, such as if they entered the room while the case-patient was having a computed tomography scan.

In summary, we conducted a thorough contact investigation of this MERS case, including a detailed characterization of the type, duration, and frequency of exposures among HCP, household, and community contacts and testing of contacts for acute disease and asymptomatic infection. We documented the absence of transmission of MERS-CoV from the first identified imported case-patient in the United States despite his having multiple contacts at home and in the hospital before the implementation of appropriate infection control procedures. In addition, our comparison of GPS-monitored contact with HCP recall of contact calls into question the accuracy of information collected by recall during a contact investigation because not all HCP reported information could be confirmed by the GPS tracer tag logs. Although factors leading to MERS-CoV transmission are likely to be complex, additional information is needed regarding the natural history of the illness, in terms of virus shedding, modes of transmission, the role of asymptomatic infections in transmission, effective infection control practices, and the length and types of exposures that do and do not lead to transmission of the virus.
